# Supplementary figures and images for: Identification of the regulatory circuit governing corneal epithelial fate determination and disease
Source: PLoS Biol. 2023 Oct 19;21(10):e3002336. doi: 10.1371/journal.pbio.3002336 (PMC10586658; doi:10.1371/journal.pbio.3002336)

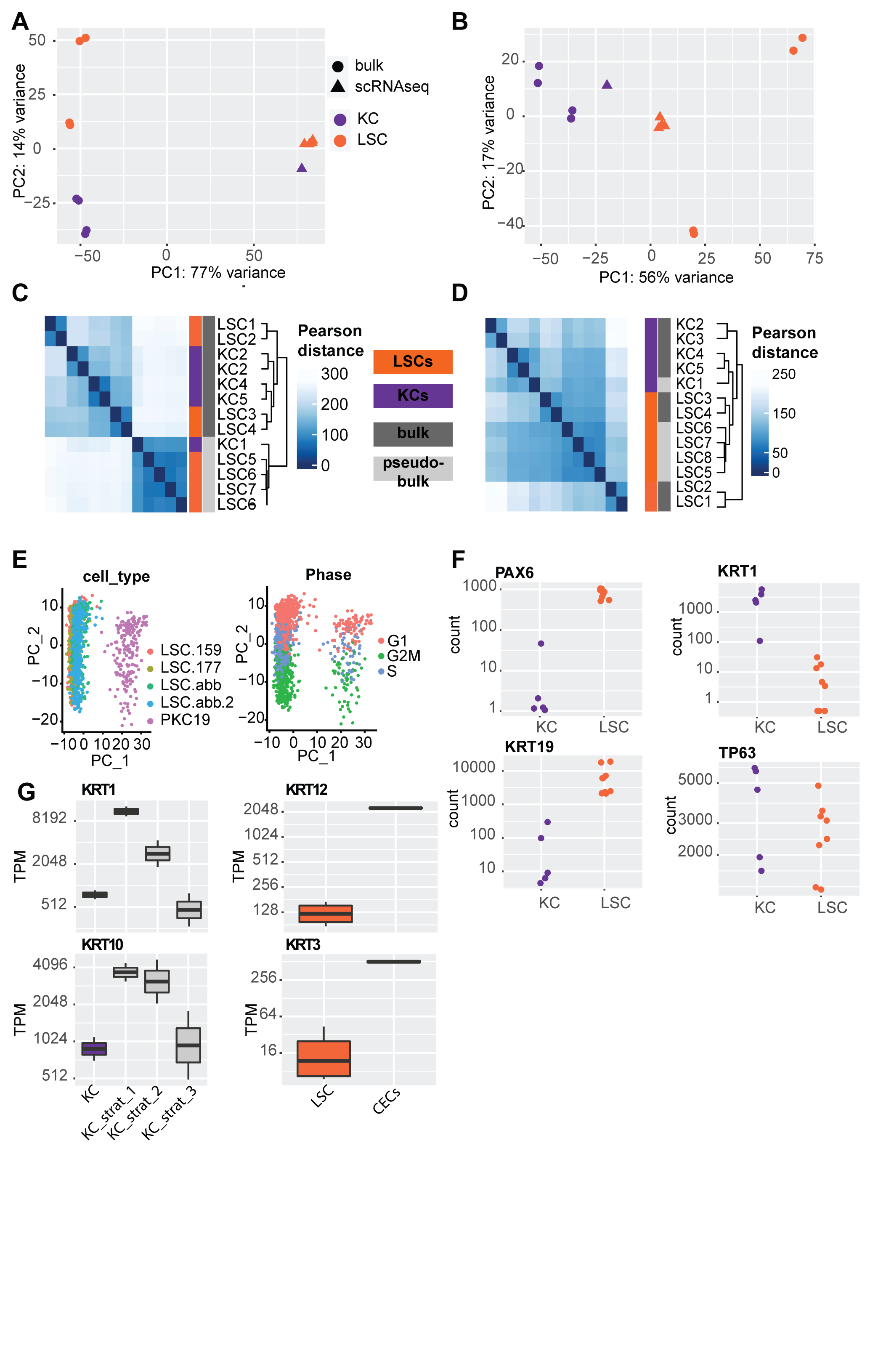

Supplement: S1 Fig — (A) PCA plot of RNA-seq samples before batch correction. (B) PCA plot after batch correction. (C) Pearson correlation matrix before batch correction. (D) Pearson correlation matrix after batch correction. (E) Umap dimensionality reduction of scRNA-seq data, visualizing the samples each cell is from on the left, and the cell cycle state on the right. (F) Gene count plot for PAX6, KRT1, KRT19, and TP63 in all KC and LSC samples. (G) TPM gene plots for KRT1, KRT10, in KC and various stratified KC samples ranging from day 2 (KC_strat_1), day 4 (KC_strat_2) and day 7 (KC_strat_3) of stratification, and of KRT12 and KRT3 in LSC and airlifted stratified cornea epithelial cells (CECs). For the underlying data, see S4 Table, GEO GSE206922, GSE206923, and GSE242995. (PNG) [file pbio.3002336.s001.png]

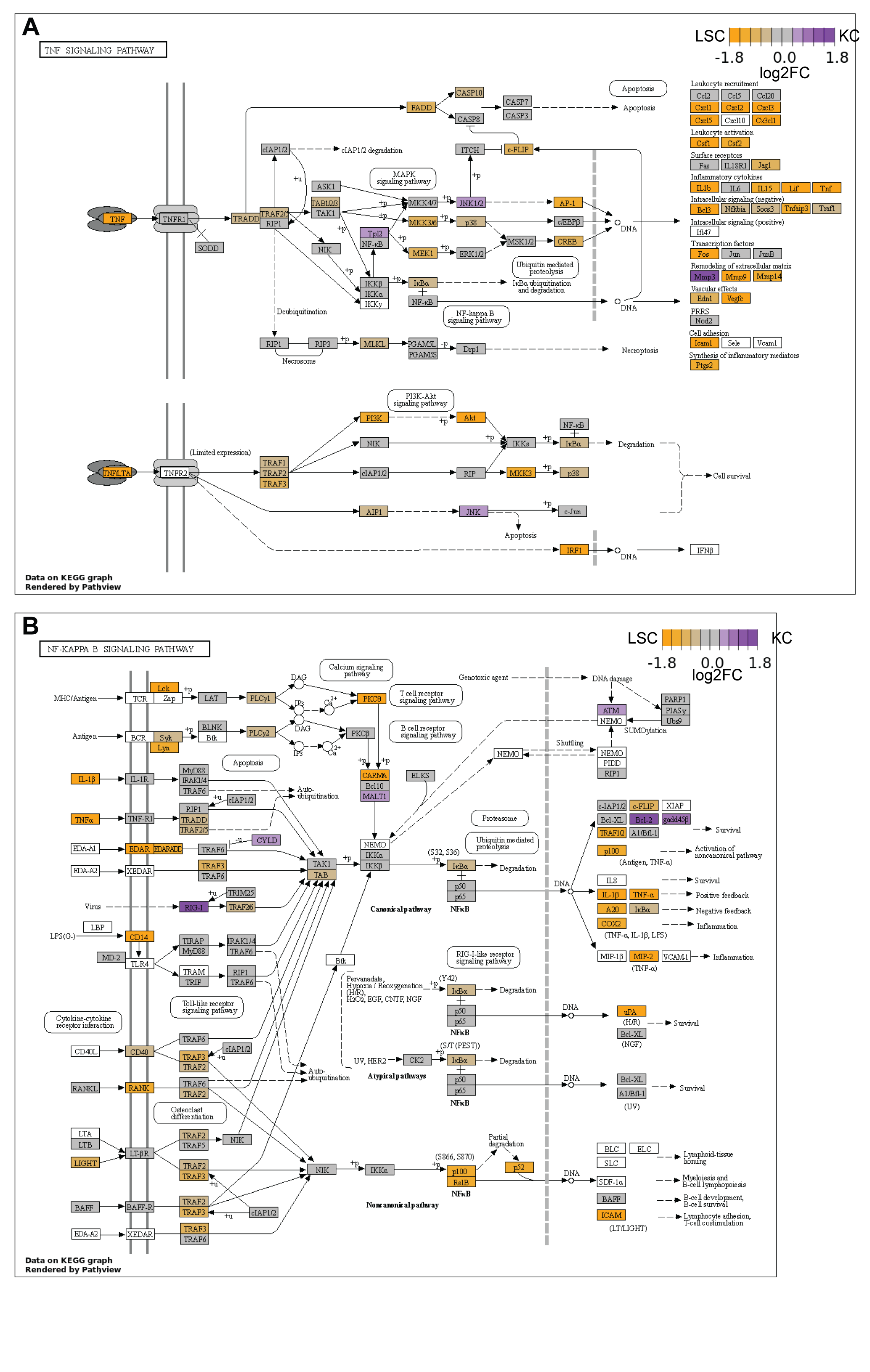

Supplement: S2 Fig — (A) TNF signaling pathway component expression FC differences between KC and LSCs. (B) NF-KAPPA B signaling pathway component expression FC differences between KC and LSCs. For the underlying data, see S4 Table, GEO GSE206922, GSE206923, and GSE242995. (PNG) [file pbio.3002336.s002.png]

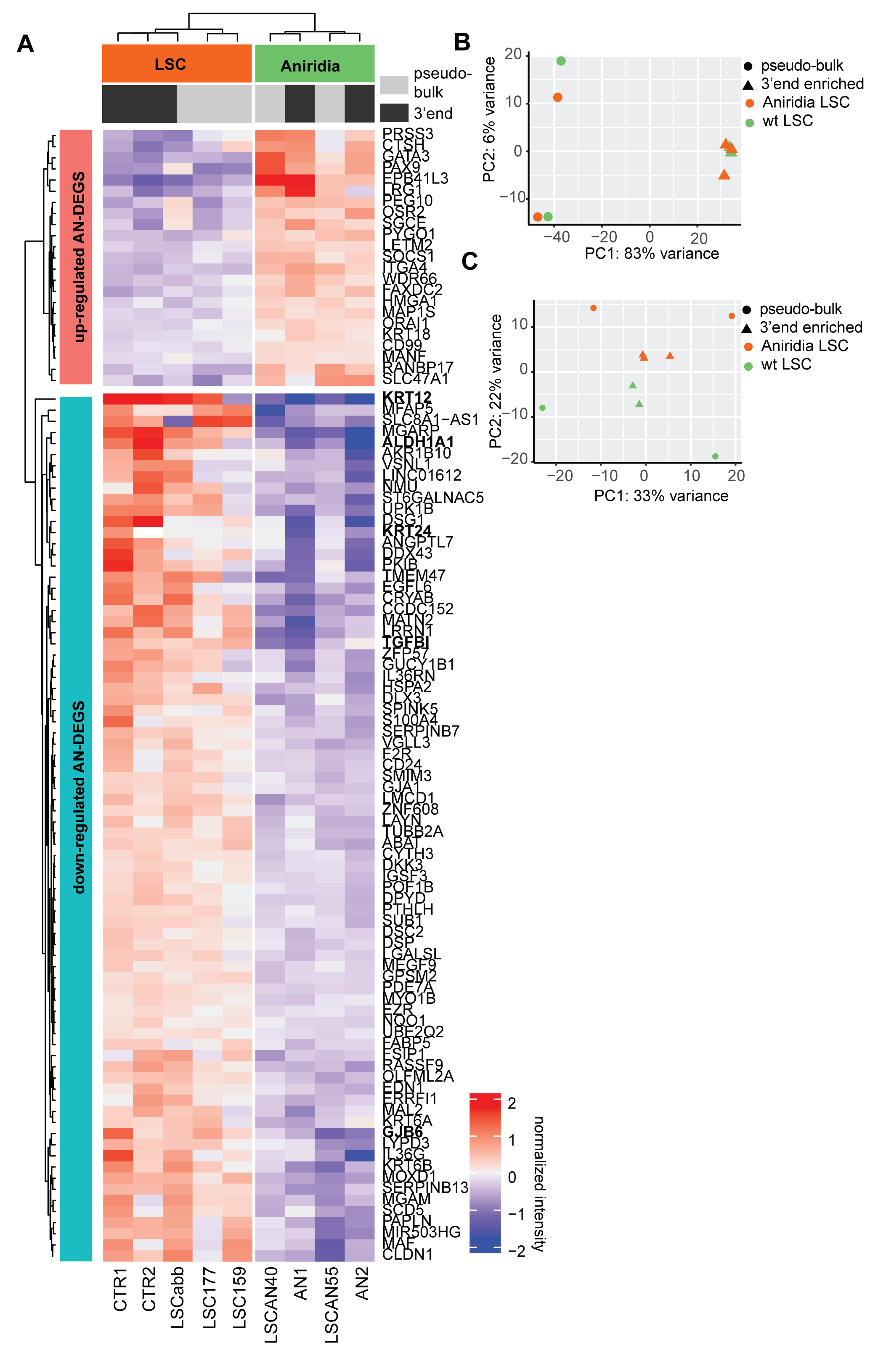

Supplement: S3 Fig — (A) Heatmap of normalized DEG expression between control and aniridia patient LSCs (adjusted pval < 0.05), using k-means clustering with 2 clusters. (B) PCA plot of RNA-seq samples before batch correction. (C) PCA plot after batch correction. For the underlying data, see S4 Table, GEO GSE206922, GSE206923, and GSE242995. (PNG) [file pbio.3002336.s003.png]

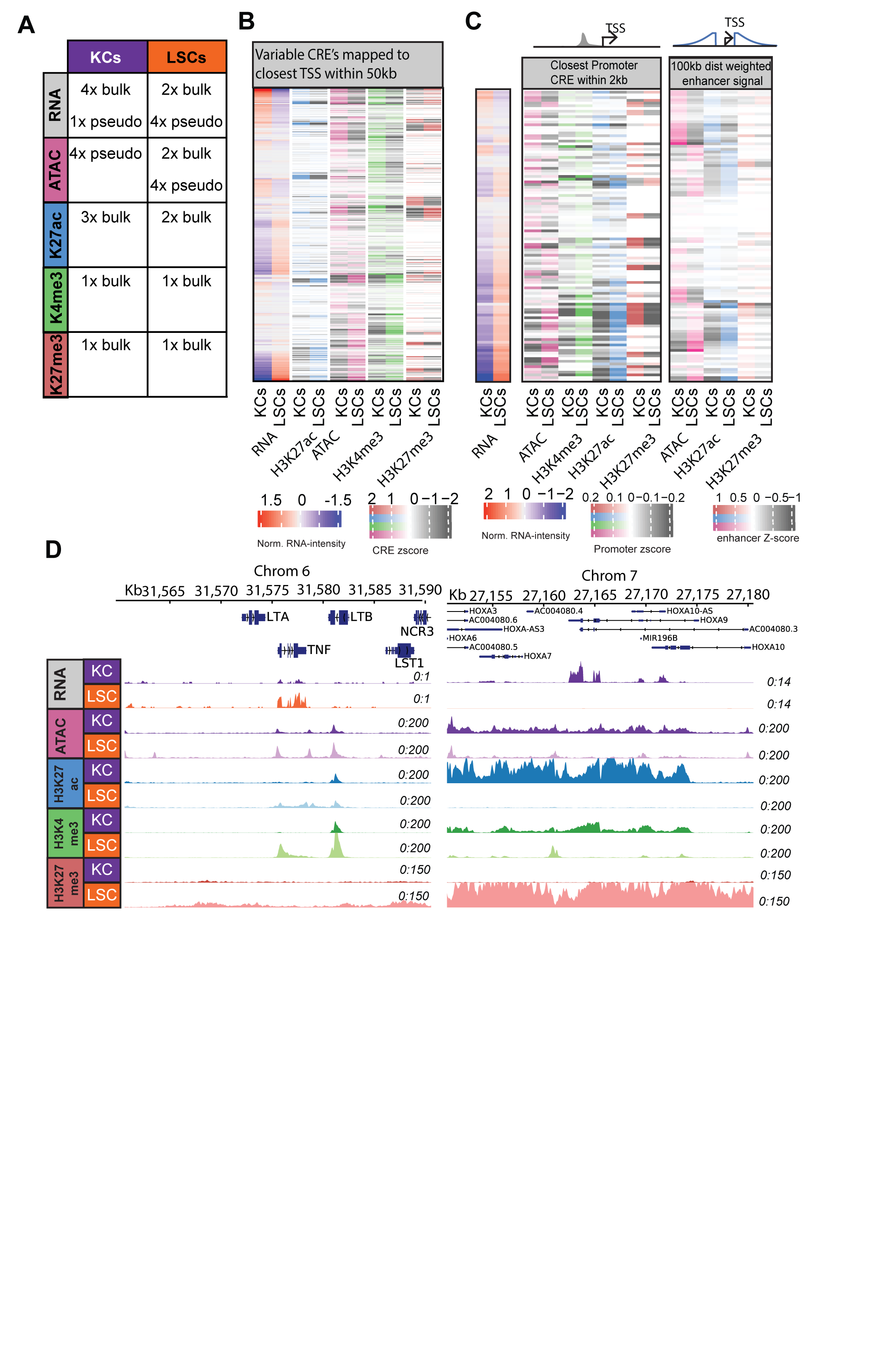

Supplement: S4 Fig — (A) Overview of data types used in our analysis. (B) Variable CREs mapped to the closest TSS within 50 kb. Zscore normalized CRE signal intensities and normalized RNA-seq intensities. (C) Heatmap of PROGENy TNF and NF-KB target genes and the Z-score of the quantile normalized histone intensity signal of the closest CRE and the distance weighted enhancer signal. (D) TNF and HOXA9 TSS loci with signals of RNA-seq, ATAC-seq, ChIP-seq of H3K27ac, H3K4me3, and H3K27me3 in KCs and LSCs. For the underlying data, see S5 Table, GEO GSE206918, GSE206920, and the trackhub in the Zenodo entry [51]. (PNG) [file pbio.3002336.s004.png]

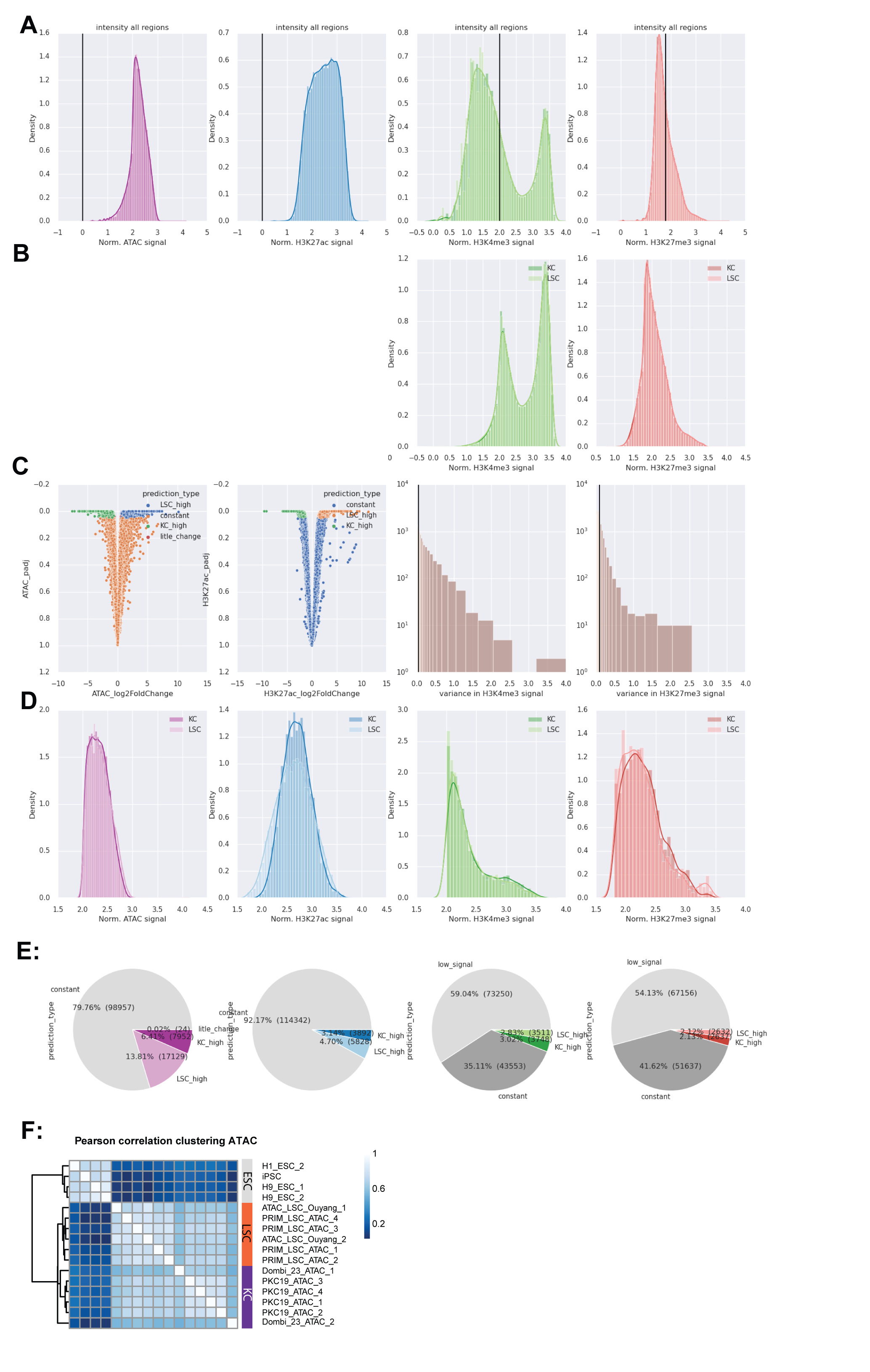

Supplement: S5 Fig — (A) Quantile normalized intensity score of all ATAC peaks for the varying histone datasets. Including cutoff value for H3K4me3 and H3K27me3. (B) Resulting intensity score for H3K4me3 and H3k27me3 regions. (C) Deseq2 volcano plot of all ATAC & H3K27ac regions. Variance with the variance cutoff for H3K4me3 and H3K27me3. (D) Resulting population of variable regions. (E) Pie chart of region type distribution. For the underlying data, see GEO GSE206918 and GSE206920. (PNG) [file pbio.3002336.s005.png]

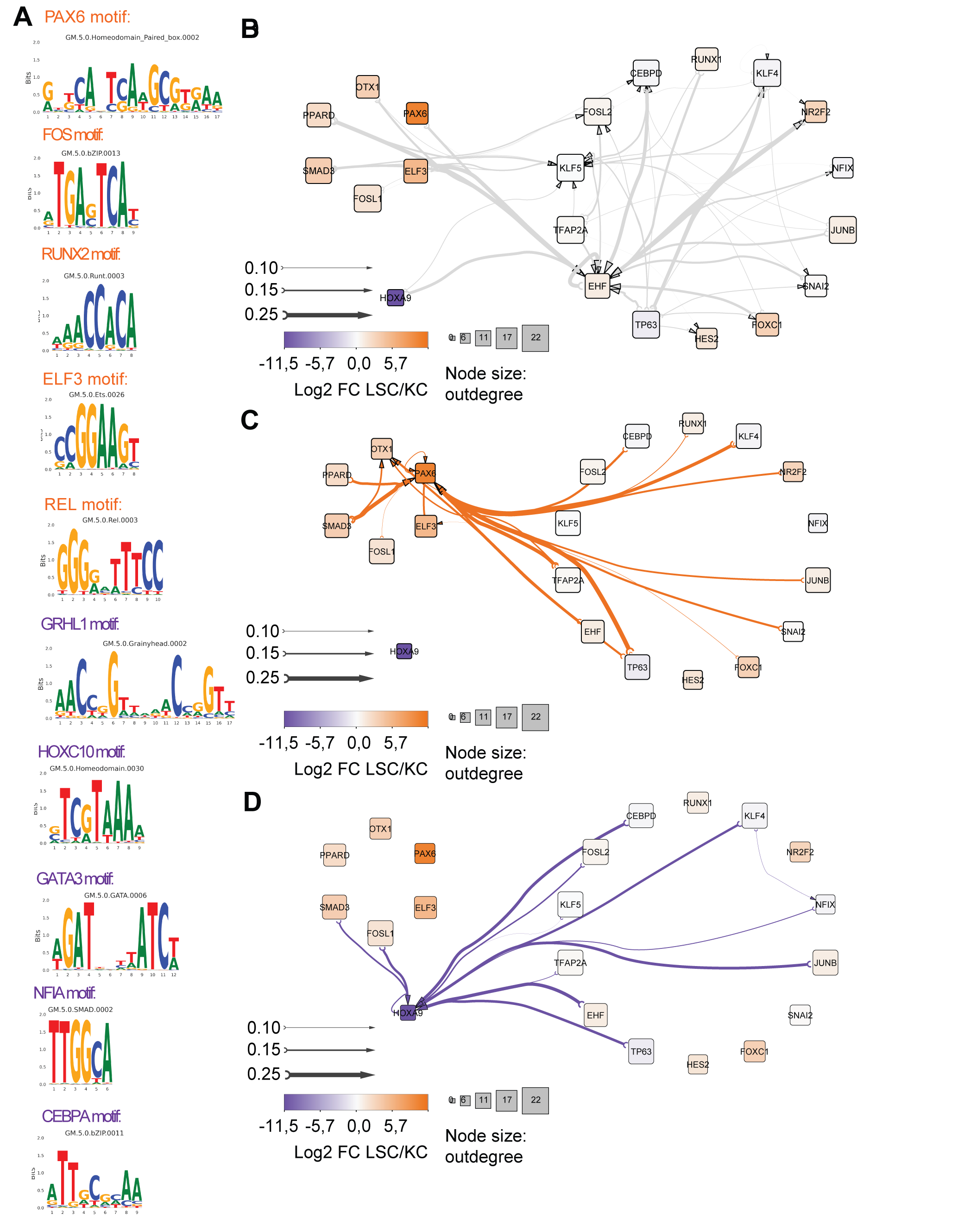

Supplement: S6 Fig — (A) Enriched motifs linked to the various TFs. (B) General epithelial interactions between TFs. Edge Width corresponds with ANANSE binding score predictions. Node color represents RNAseq fold change between LSC and KCs, while node size represents outdegree. (C) Similar to B but with all the LSC-specific interactions. (D) Similar to B but with all the KC-specific interactions. For underlying data, see the Zenodo entry [51]. (PNG) [file pbio.3002336.s006.png]

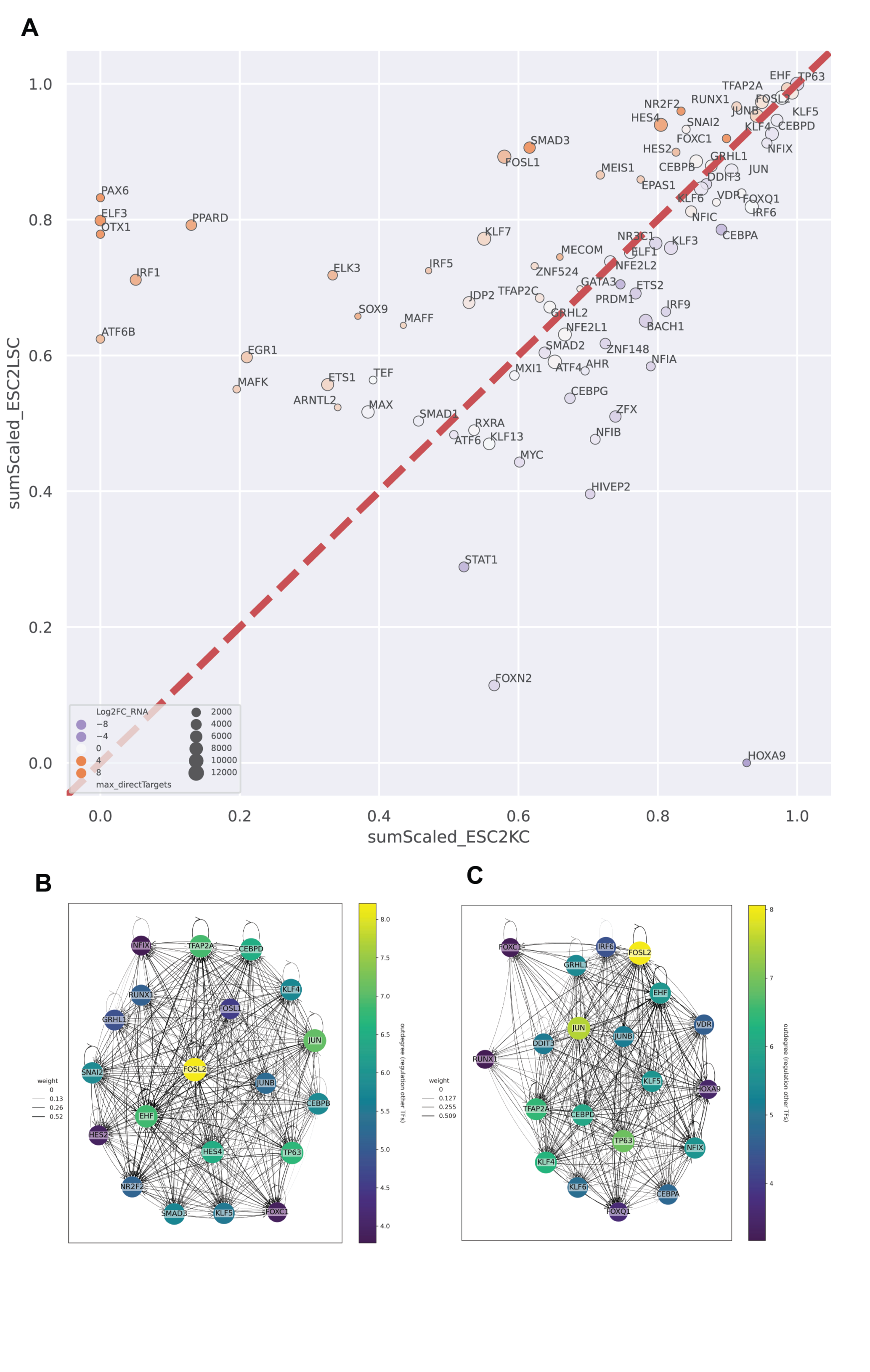

Supplement: S7 Fig — (A) ANANSE influence of ESC to KC (x-axis) and ESC to LSC (y-axis), circle size represents a maximum number of target genes in both comparisons. The circle color represents log2FC between LSC/KC. (B) ESC-LSC top TF interaction network generated by ANANSE. (C) ESC-KC top TF interaction network generated by ANANSE. For the underlying data, see the Zenodo entry [51]. (PNG) [file pbio.3002336.s007.png]

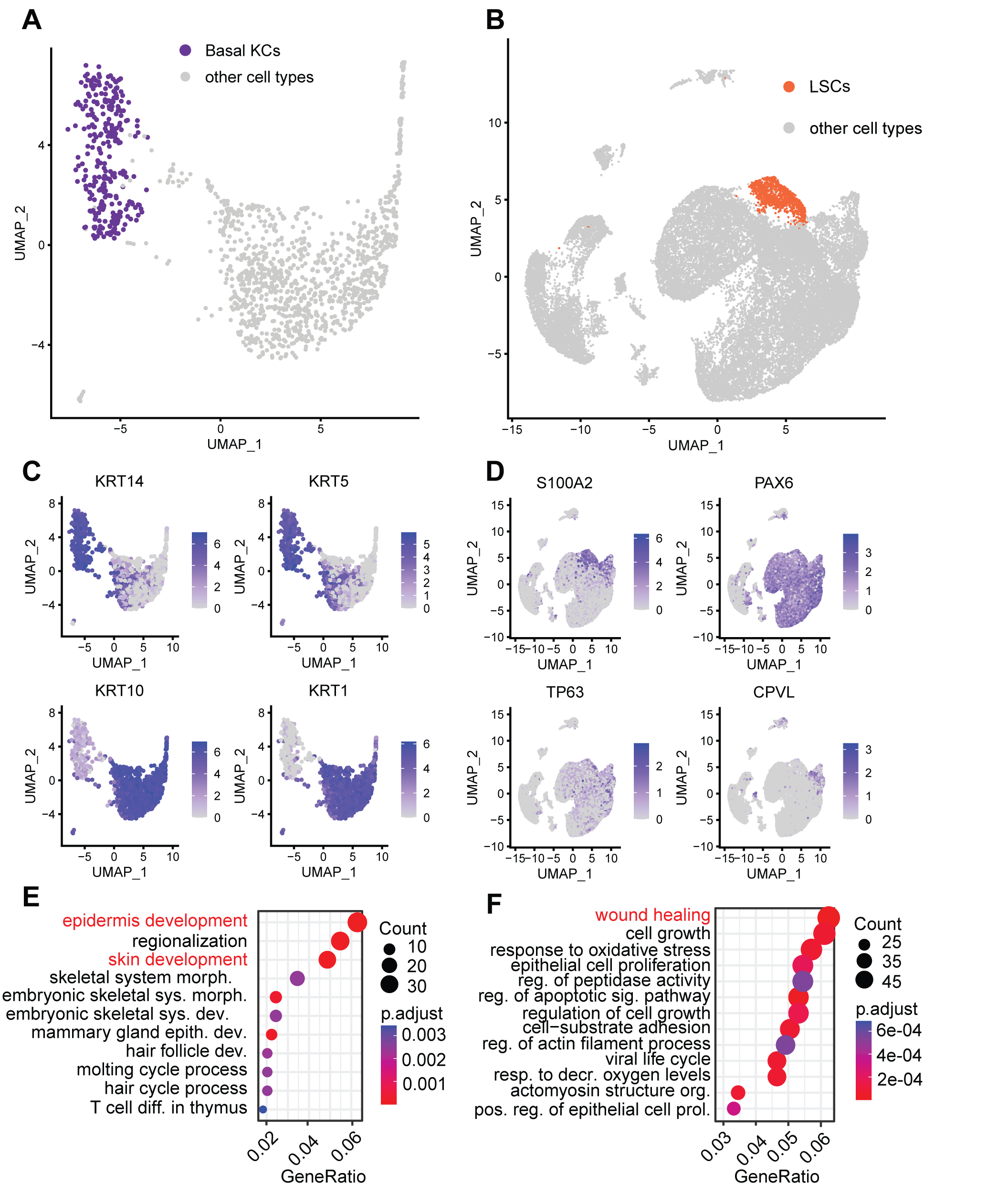

Supplement: S8 Fig — (A) Umap of epidermal scRNAseq dataset of Atwood and colleagues. Basal KC cluster used for validation is highlighted. (B) Umap of scCornea atlas of Collin and colleagues, basal LSC cluster used for validation is highlighted. (C) Marker gene expression used to select the basal KCs cluster. (D) Marker gene expression used to select the basal LSC cluster. (E) GO term enrichment of the basal-KC high DEGS enriched vs. the human genome as a background and simplified using simplify. (F) GO term enrichment of the basal-LSCs high DEGS enriched versus the human genome as a background and simplified using simplify. For the underlying data, see GEO GSE155683 and GSE147482 [55,56]. (PNG) [file pbio.3002336.s008.png]

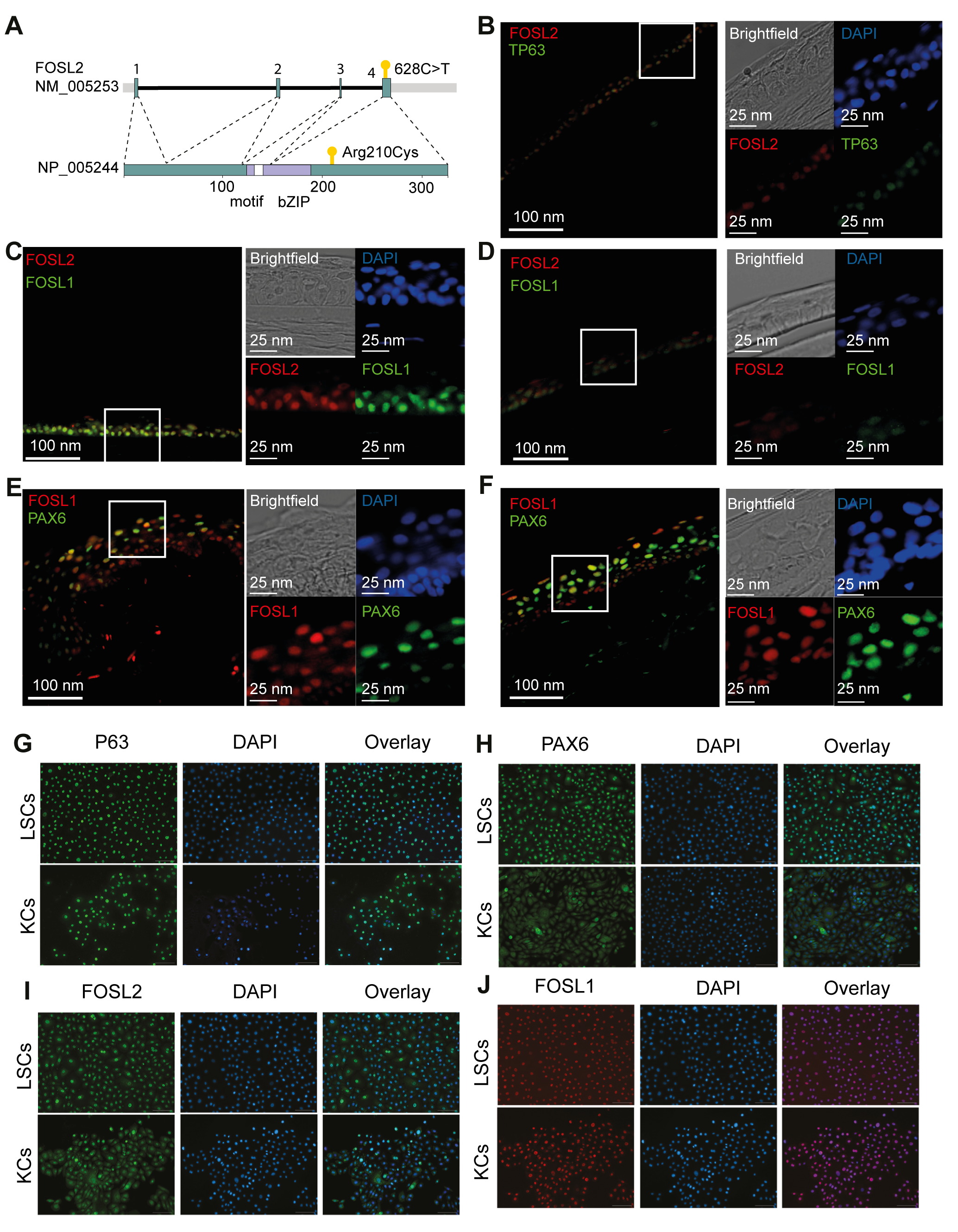

Supplement: S9 Fig — (A) Overview of the FOSL2 transcript and protein, with the location of the variant of unknown significance. (B) FOSL2 and TP63 staining of the central cornea. (C) FOSL1 and FOSL2 staining of the peripheral cornea (D) FOSL1 and FOSL2 staining of the central cornea. (E) FOSL2 and PAX6 staining of the peripheral cornea. (F) FOSL1 and FOSL2 staining of the central cornea. (G–J) Immunocytochemistry analysis of fixed LSCs and KCs of transcription factors p63 (E), PAX6 (F), FOSL2 (G), and FOSL1 (H). Predicted general TFs p63, FOSL1, and FOSL2 are detected in the nuclei of both cell types while PAX6 is only detected in LSCs. Note that some non-nuclear signal is found in KCs stained with PAX6 but that is considered autofluorescence. DAPI staining (blue) depicts cell nuclei. Scale bar, 100 μm. (PNG) [file pbio.3002336.s009.png]

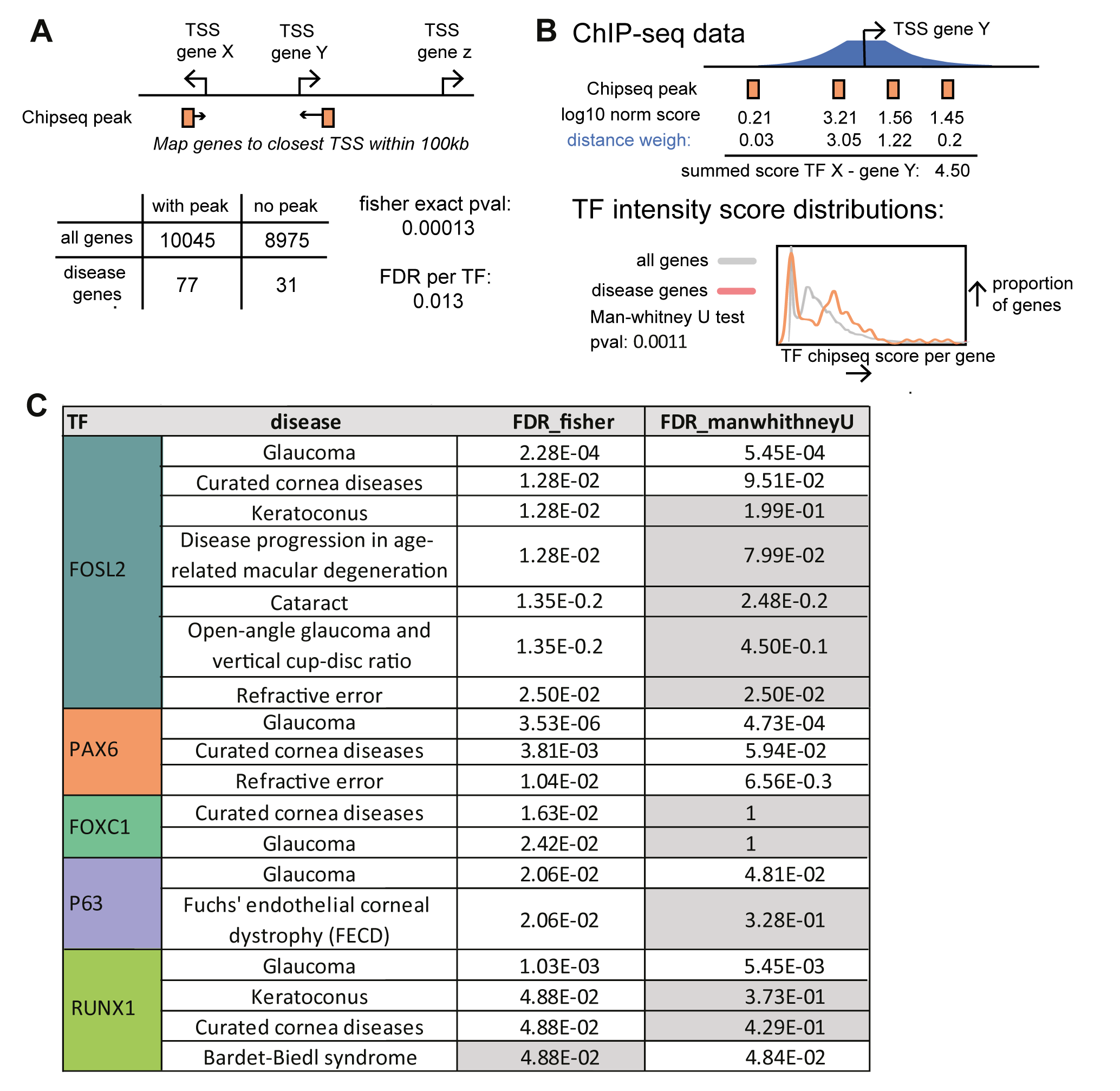

Supplement: S10 Fig — (A) Approach for distance weighing and merging of TF ChIP-seqs per TF. This resulted in a TF-disease gene score distribution that was compared to the distribution of all genes with a one-sided Mann–Whitney U test. (B) Approach for linking the ChIP-seq peaks to the closest gene TSS, after which enrichment for disease genes was tested with a Fisher exact test. (C) FDR values of the significant enriched TFs resulting from the ChIP-seq Mann–Whitney U tests and the Fisher exact test. Significant for FDR < 0.1. For the underlying data, see S7 Table, GEO GSE206920, GSE236440, and GSE156272. (PNG) [file pbio.3002336.s010.png]

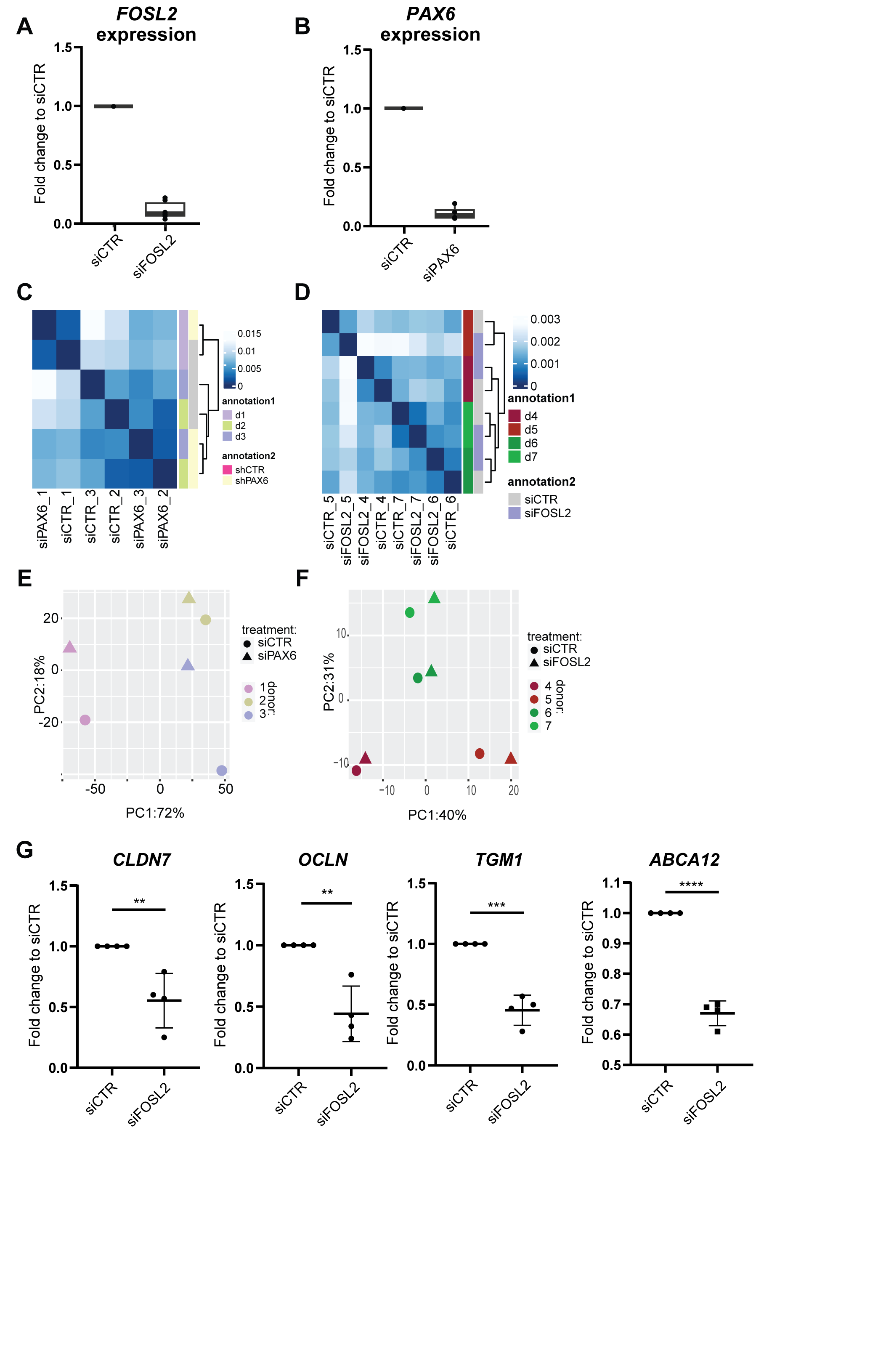

Supplement: S11 Fig — (A) qPCR validation FOSL2 knockdown the data underlying this figure can be found at S10 Table. (B) qPCR validation PAX6 knockdown the data underlying this figure can be found at S10 Table. (C) Pearson correlation matrix siCTR and siPAX6 samples. (D) Pearson correlation matrix siCTR and siFOSL2 samples. (E) PCA plot of RNAseq siPAX6 samples. (F) PCA plot of RNA-seq siFOSL2 samples. (G) Transcripts CLDN7, OCLN, TGM1, and ABCA12 were measured in control LSCs (CTRL) and FOSL2 siRNA-knock down (FOSL2 KD) samples (n = 4). Values represent fold change difference of FOSL2 KD to their respective CTRL and were normalized to internal housekeepers GAPDH and ACTB (* pval<0.05, ** pval<0.01, *** pval<0.001, unpaired t-test analysis). For the underlying data, see S10 Table, GEO GSE242990, and GSE236440. (PNG) [file pbio.3002336.s011.png]
