# Supplementary material for: Identification of the regulatory circuit governing corneal epithelial fate determination and disease
Source: PLoS Biol. 2023 Oct 19;21(10):e3002336. doi: 10.1371/journal.pbio.3002336 (PMC10586658; doi:10.1371/journal.pbio.3002336)
Supplement: S1 Table — Overview of all RNA-seq datasets generated and used, including datatype, origin, medium condition, and GEO accession number. (DOCX) [file pbio.3002336.s012.docx]

| **dataset** | **name:** | **datatype** | **origin** | **medium** | **GEO number** |
| --- | --- | --- | --- | --- | --- |
| KC1 | PKC19 | pseudobulk | donor skin | KBM | GSM6266912 |
| KC2 | PKC19 | bulk | donor skin | KBM | GSM2597280 |
| KC3 | Dombi23 | bulk | donor skin | KBM | GSM2597284 |
| KC4 | PKC19 | bulk | donor skin | KBM | GSM6266906 |
| KC5 | PKC19 | bulk | donor skin | KBM | GSM6266907 |
| LSC1 | LSC_ouyang | bulk | postmortem donor | F12 | GSM4728059 |
| LSC2 | LSC_ouyang | bulk | postmortem donor | F12 | GSM4728060 |
| LSC3 | LSCaberdam | bulk | postmortem donor | KSFM | GSM6266904 |
| LSC4 | LSCaberdam | bulk | postmortem donor | KSFM | GSM6266905 |
| LSC5 | LSC159 | pseudobulk | postmortem donor | KSFM | GSM6266908 |
| LSC6 | LSC177 | pseudobulk | postmortem donor | KSFM | GSM6266909 |
| LSC7 | LSCaberdam | pseudobulk | postmortem donor | KSFM | GSM6266910 |
| LSC8 | LSCaberdam | pseudobulk | postmortem donor | KSFM | GSM6266911 |
| CTR1 | CTR1 | 3’ bulk | postmortem donor | KSFM | GSM3093490 |
| CTR2 | CTR2 | 3’ bulk | postmortem donor | KSFM | GSM3093491 |
| AN1 | AN1 | 3’ bulk | keratoplasty | KSFM | GSM3093492 |
| AN2 | AN2 | 3’ bulk | keratoplasty | KSFM | GSM3093493 |
| Aniridia3 | AN40 | pseudobulk | keratoplasty | KSFM | GSM7776904 |
| Aniridia4 | AN55 | pseudobulk | keratoplasty | KSFM | GSM7776905 |
| scCornea | Cornea tissue | Single-cell | postmortem donor |  | GSE155683 |
| scEpidermis | Epidermal tissue | Single-cell | postmortem donor |  | GSE147482 |
| ESC1 | H1 | 3’ bulk | cell line |  | GSM915329 |
| ESC2 | H11 | 3’ bulk | cell line |  | GSM958733 |
| KC_strat_1 | PKC19 | bulk | donor skin | KBM | GSM2597281 |
| KC_strat_1 | PKC19 | bulk | donor skin | KBM | GSM2597285 |
| KC_strat_2 | PKC19 | bulk | donor skin | KBM | GSM2597282 |
| KC_strat_2 | PKC19 | bulk | donor skin | KBM | GSM2597286 |
| KC_strat_3 | PKC19 | bulk | donor skin | KBM | GSM2597283 |
| KC_strat_3 | PKC19 | bulk | donor skin | KBM | GSM2597287 |
| CECs | LSC_ouyang | bulk | postmortem donor | F12 | GSM4711910 |
| CECs | LSC_ouyang | bulk | postmortem donor | F12 | GSM4711911 |
