# Supplementary material for: Identification of the regulatory circuit governing corneal epithelial fate determination and disease
Source: PLoS Biol. 2023 Oct 19;21(10):e3002336. doi: 10.1371/journal.pbio.3002336 (PMC10586658; doi:10.1371/journal.pbio.3002336)
Supplement: S2 Table — Overview of all ATAC-seq datasets generated and used, including datatype, origin, medium condition, and GEO accession number. (DOCX) [file pbio.3002336.s013.docx]

| **dataset** | **name:** | **datatype** | **origin** | **medium** | **GEO number** |
| --- | --- | --- | --- | --- | --- |
| KC1 | Dombi23 | bulk | donor skin | KBM | GSM6266861 |
| KC2 | Dombi23 | bulk | donor skin | KBM | GSM6266862 |
| KC3 | PKC19 | pseudobulk | donor skin | KBM | GSM6266863 |
| KC4 | PKC19 | pseudobulk | donor skin | KBM | GSM6266864 |
| KC5 | PKC19 | pseudobulk | donor skin | KBM | GSM6266865 |
| KC6 | PKC19 | pseudobulk | donor skin | KBM | GSM6266866 |
| LSC1 | LSCaberdam | pseudobulk | postmortem donor | KSFM | GSM6266867 |
| LSC2 | LSCaberdam | pseudobulk | postmortem donor | KSFM | GSM6266868 |
| LSC3 | LSC 159 | pseudobulk | postmortem donor | KSFM | GSM6266869 |
| LSC4 | LSCaberdam | bulk | postmortem donor | KSFM | GSM6266870 |
| LSC5 | LSC_Ouyang | Bulk | postmortem donor | KSFM | GSM4728093 |
| LSC6 | LSC_Ouyang | Bulk | postmortem donor | KSFM | GSM4728094 |
| ESC1 | H1 | Bulk |  | HSFEM | GSM2400260 |
| ESC1 | H1 | bulk |  | HSFEM | GSM2400261 |
