# Supplementary material for: Identification of the regulatory circuit governing corneal epithelial fate determination and disease
Source: PLoS Biol. 2023 Oct 19;21(10):e3002336. doi: 10.1371/journal.pbio.3002336 (PMC10586658; doi:10.1371/journal.pbio.3002336)
Supplement: S3 Table — Overview of all TF binding datasets generated and used, including datatype, origin, medium condition, and GEO accession number. (DOCX) [file pbio.3002336.s014.docx]

| **dataset** | **Cell Type** | **name:** | **datatype** | **origin** | **Medium** | **GEO number** |
| --- | --- | --- | --- | --- | --- | --- |
| KC1_H3K27ac | KC | HKC1 | H3K27ac | donor skin | KBM | GSM1446919 |
| KC2_H3K27ac | KC | Dombi23 | H3K27ac | donor skin | KBM | GSM6266884 |
| LSC1_H3K27ac | LSC | LSC JQ | H3K27ac | postmortem donor | KSFM | GSM6266886 |
| LSC2_H3K27ac | LSC | LSC_Ouyang | H3K27ac | postmortem donor | F12 | GSM4728063 |
| LSC3_H3K27ac | LSC | LSC_Ouyang | H3K27ac | postmortem donor | F12 | GSM4728064 |
| ESC1 H3K27ac | ESCs | H1 | H3K27ac |  |  | GSM466732 |
| ESC2 H3K27ac | ESCs | H1 | H3K27ac |  |  | GSM663427 |
| ESC3 H3K27ac | ESCs | H1 | H3K27ac |  |  | GSM733718 |
| KC1_H3K27me3 | KC | Dombi23 | H3K27me3 | donor skin | KBM | GSM2597292 |
| LSC1_H3K27me3 | LSC | LSC_JQ | H3K27me3 | postmortem donor | KSFM | GSM6266887 |
| LSC2_H3K27me3 | LSC | LSC_Ouyang | H3K27me3 | postmortem donor | F12 | GSM4728069 |
| LSC3_H3K27me3 | LSC | LSC_Ouyang | H3K27me3 | postmortem donor | F12 | GSM4728070 |
| KC1_H3K4me3 | KC | Dombi23 | H3K4me3 | donor skin | KBM | GSM2597288 |
| LSC1_H3K4me3 | LSC | LSC JQ | H3K4me3 | postmortem donor | KSFM | GSM6266881 |
| LSC2_H3K4me3 | LSC | LSC_Ouyang | H3K4me3 | postmortem donor | F12 | GSM4728067 |
| LSC3_H3K4me3 | LSC | LSC_Ouyang | H3K4me3 | postmortem donor | F12 | GSM4728068 |
| LSC1 P63 1 | LSC | LSC JQ | P63 | postmortem donor | KSFM | GSM6266882 |
| LSC2 P63 2 | LSC | LSC JQ | P63 | postmortem donor | KSFM | GSM6266883 |
| KC1 P63 | KC | HKC1 | P63 | donor skin | KBM | SRR1528616 |
| KC2 P63 | KC | Dombi23 | P63 | donor skin | KBM | GSM6266880 |
| LSC1 RUNX1 | LSC | LSC_Ouyang | RUNX1 | postmortem donor | F12 | GSM4728087 |
| LSC2 RUNX1 | LSC | LSC_Ouyang | RUNX1 | postmortem donor | F12 | GSM4728088 |
| LSC1 PAX6 | LSC | LSC_Ouyang | PAX6 | postmortem donor | F12 | GSM4728089 |
| LSC2 PAX6 | LSC | LSC_Ouyang | PAX6 | postmortem donor | F12 | GSM4728090 |
| LSC1 SMAD3 | LSC | LSC_Ouyang | SMAD3 | postmortem donor | F12 | GSM4728091 |
| LSC2 Smad3 | LSC | LSC_Ouyang | SMAD3 | postmortem donor | F12 | GSM4728092 |
| LSC1 FOXC1 | LSC | LSC_Ouyang | FOXC1 | postmortem donor | F12 | GSM4711922 |
| LSC2 FOXC1 | LSC | LSC_Ouyang | FOXC1 | postmortem donor | F12 | GSM4711923 |
| LSC1 FOSL2 | LSC | LSC_CNR | CUT&RUN_FOSL2 | postmortem donor | KSFM | GSM7544341 |
| LSC2 FOSL2 | LSC | LSC_CNR | CUT&RUN_FOSL2 | postmortem donor | KSFM | GSM7544342 |
