# Supplementary material for: Identification of the regulatory circuit governing corneal epithelial fate determination and disease
Source: PLoS Biol. 2023 Oct 19;21(10):e3002336. doi: 10.1371/journal.pbio.3002336 (PMC10586658; doi:10.1371/journal.pbio.3002336)
Supplement: S8 Table — Overview of all the HP terms included and excluded, and the number of GEL probands. (DOCX) [file pbio.3002336.s019.docx]

| **Included HP (daughter) terms of Corneal opacity** |  | GEL probands |
| --- | --- | --- |
| Corneal opacity | HP:0007957 | 12 |
| Sclerocornea | HP:0000647 | 8 |
| Corneal scarring | HP:0000559 | 3 |
| Opacification of the corneal stroma | HP:0007759 | 3 |
| Central posterior corneal opacity | HP:0008511 | 2 |
| Generalized opacification of the cornea | HP:0011494 | 2 |
| Central opacification of the cornea | HP:0011493 | 1 |
| Band keratopathy | HP:0000585 | 1 |
| Corneal crystals | HP:0000531 | 1 |
| Kayser-Fleischer ring | HP:0200032 | 0 |
| Opacification of the corneal epithelium | HP:0007727 | 0 |
| Subepithelial corneal opacities | HP:0008039 | 0 |
| Peripheral opacification of the cornea | HP:0008011 | 0 |
| Punctate opacification of the cornea | HP:0007856 | 0 |
| Total included: |  | 33 |
|  |  |  |
| **Excluded terms** |  |  |
| Corneal arcus | HP:0001084 | 60 |
| Central corneal dystrophy | HP:0007881 | 4 |
| Mosaic central corneal dystrophy | HP:0100690 | 0 |
| Nodular corneal dystrophy | HP:0007827 | 0 |
| Crystalline corneal dystrophy | HP:0007760 | 1 |
| Crystalline corneal dystrophy | HP:0007760 | 1 |
| Total excluded: |  | 66 |
