# Supplementary material for: Identification of the regulatory circuit governing corneal epithelial fate determination and disease
Source: PLoS Biol. 2023 Oct 19;21(10):e3002336. doi: 10.1371/journal.pbio.3002336 (PMC10586658; doi:10.1371/journal.pbio.3002336)
Supplement: S9 Table — Overview of the FOSL2 variant info and the pathogenic prediction scores. (DOCX) [file pbio.3002336.s020.docx]

| **FOSL2(NM_005253.4):c.628C>T. (p.Arg210Cys)** | | |
| --- | --- | --- |
| **variant info** | GenBank | NM_005253.4 |
|  | Uniprot | P15408 |
|  | genomicDNA-hg38 | 2-28412095-C-T |
|  | cDNA | c.628C>T |
|  | AminoAcid | 210 |
|  | reference AA | R |
|  | Alternative AA | c.628C>T |
|  | gnomAD allele freq | 0.0000922 |
| ACMG/AMG guidelines^123^ classification | variant of uncertain significance | |
| **prediction tool** | **prediction score** | **prediction** |
| SIFT | 0.035 | **Damaging** |
| SIFT4G | 0.034 | **Damaging** |
| PrimateAI | 0.73 | **Pathogenic** |
| MutationTaster | 0.99999 | **Disease Causing** |
| FATHMM-XF | 0.90467 | **Pathogenic** |
| List-S2 | 0.886611 | **Damaging** |
| Meta-RNN | 0.5091318 | **Damaging** |
| M-CAP | 0.096 | Possibly Pathogenic |
| Poly-phen2 | 0.096 | Probably Damaging |
| MutPred | 0.103 | Benign |
| Provean | -1.5 | Neutral |
| FATHMM | -0.17 | Tolerated |
| CADD | 27.1 |  |
| R210C | in VERY LOW Alpha Fold predicted region (disordered) |  |
