# Supplementary material for: Identification of the regulatory circuit governing corneal epithelial fate determination and disease
Source: PLoS Biol. 2023 Oct 19;21(10):e3002336. doi: 10.1371/journal.pbio.3002336 (PMC10586658; doi:10.1371/journal.pbio.3002336)
Supplement: Attachment — Submitted filename: 132063_1_rebuttal_2605994_rqkzgd.pdf [file pbio.3002336.s022.pdf]

Revision Plan

Manuscript number: RC-2022-01765
Corresponding author(s): Dr. Huiqing Zhou (Radboud University)

1. General Statements [optional]

We like to thank the editor and reviewers for their constructive comments and suggestions for improving the manuscript. We here address the comments point-by-point using the template of the revision plan.

2. Description of the planned revisions

Reviewer #1:
2. While the study is complete and describes well, a strong conclusion, including validation of the role of some TFs such as FOSL2 through knock out experiments in model organisms or cell culture will elevate the paper more (optional).

To address this point, we will perform siRNA knockdown experiments of TFs identified in our study, including FOSL2, in primary LSCs, and examine the transcriptional consequences of knocking down these TFs by RNA-seq or RT-qPCR analyses.

Reviewer #2:
1. The findings provide an overview of transcriptional regulators and targets in two essential tissues. This is a valuable tool for future discoveries regarding the processes governing cell differentiation and those involved in the disease mechanism of the cornea. Although the presented predictions are interesting, what is missing is an examination of the functional significance of the findings.

Indeed, we fully agree with the reviewer that additional functional examinations are important and relevant and would strengthen the manuscript. We propose to do the following functional analyses to further demonstrate the importance of the key TFs.
1) Immunostaining of the key TFs in the human cornea and in LSCs and KCs.
2) As described above, we will perform siRNA experiments for key TFs identified in our study, followed by RNA-seq or RT-qPCR analysis, to assess the transcriptional program controlled by these key TFs.
3) The gene regulatory network controlled by these tested TFs will be analysed, to examine the interplay of these TFs in transcriptional regulation and in cell fate determination.

Reviewer #2:
7. Also, the findings indicate an interaction between FOXL2 and other TFS is important for maintaining the corneal epithelium. These interesting predictions indicate an important role for FoxL2 in corneal function. It would be important to verify these predictions by experimental

studies, for example, by presenting the association of FOXL2 with the predicted co-factors and presenting data on the effect of the identified mutation on FOXL2 transcriptional activity.

We assume that Reviewer #2 refers to FOSL2 instead of FOXL2. We agree with this reviewer's suggestion to functionally address the importance of FOSL2 in the cornea. In order to answer this, we plan to perform FOSL2 staining and FOSL2 siRNA knockdown in LSCs, followed by RNA-seq, as described above. This will show the FOSL2 importance in LSCs and in cornea, and will identify the affected downstream gene networks.

Regarding the clinical effect of the specific *FOSL2* variant reported in our study, we agree that functional validation would strengthen our work even more. We believe that the main message of the study is the use of integrative omics analyses to uncover new transcription factors involved in corneal and limbal fates, and to highlight new candidate genes in corneal disease. Therefore we feel that the disease mechanism behind the specific *FOSL2* variant, albeit interesting, is beyond the scope of this study. Nonetheless, we reinforced the pathogenicity of the variant with various in silico prediction platforms (supplementary table 9). Interestingly, a recent study reported that FOSL2 truncating mutations are involved in a new syndrome with ectodermal defects and cataract. This is in line with our findings that FOSL2 is an important shared TF in both LSCs and KCs, and strengthens the predicted role of FOSL2 in the epithelium of the eye and associated diseases. We have included additional discussion on this study in the Discussion line 662-668.

3. Description of the revisions that have already been incorporated in the transferred manuscript

Reviewer #2:
2. *In Figure 1, the authors compare the transcriptome and epigenome (ATAC-seq and histone modifications) of basal KCs from skin donors and cultured LSCs established from limbal biopsies. The authors should clarify the source of the cells in the published studies - specifically, why more data were needed and if these were comparable to their datasets.*

We have included the cell sources and cultured conditions from the published studies and added additional columns in the supplementary tables 1, 2 and 3. Briefly, LSC publicly available samples were extracted from post-mortem cornea and cultured in DMEM/F12, or KSFM.

Regarding the questions on the necessity of incorporating more data, our reasoning was two-fold. First of all, we have taken an integrated approach to perform our analysis, using both our own and publicly available datasets. We see this as a strength, as the most important differences between cell types that determine cell fates should be consistent with cells generated from different donors and labs. Second, we choose to generate more data in our own lab in order to make sure to have comparisons without the influence of technical differences between publicly available datasets. We include text about this approach in the Discussion (lines) 578-580. Furthermore, to show that the datasets we used are consistent and can be integrated, we have performed a PCA correlation analysis for the RNA-seq analysis (supplementary figures 1A&1B lines 834-837), and added a spearman correlation analysis for the ATAC-seq datasets (supplementary figure 5F & lines 818-820). Both indicated clear biological signal similarities between cell types across different labs and techniques.

Reviewer #2:
3.Next, they compared the transcriptomes of LSCs and KCs to the transcription profile of LSCs from two aniridia patients and control. They need to specify the stage of the donors' disease and provide details on the control samples.

Both aniridia samples were from patients of stage 4 on the Lagali Stages (Lagali, N. et al. (2020) 'Early phenotypic features of aniridia-associated keratopathy and association with PAX6 coding mutations', The Ocular Surface, 18(1), pp. 130–140. We have included this information in Material and Methods (lines 738-739). For healthy control cells, no information regarding the stage and gender is available, as they are from anonymous individuals. We added more information on the aniridia and control samples regarding the culture conditions and passage numbers (lines 747:750).

Reviewer #2:
4. In addition, and as indicated above, when combining published datasets, one should clarify whether the methods of collecting/growing the cells and the disease stage are comparable. This

is important with samples from aniridia as it is unclear if the patient LSCs survive the isolation or if other cells take over.

Healthy LSC cells used in the direct comparison with aniridia LSC cells were grown using the same expansion and culture conditions. Furthermore, the method of culture, extraction and expansion between the earlier published aniridia cell data and our data are exactly the same (lines 735:750). As described above, we have included the cell sources from the aniridia samples, and added additional columns in the supplementary table 1.

Reviewer #2:
6. It would be valuable to the community if the presented data were also provided online in a web tool so that CRE activity or gene expression could be easily examined.

We have expanded the UCSC track hub to highlight the identified variable CRE elements. This will enable a searchable tool for differential CREs close to genes of interest between KCs and LSCs. We have furthermore added a sentence to explicitly mention the presence of this track hub in the result section (lines 248-249).

4. Description of analyses that authors prefer not to carry out

Reviewer #1:

1. *By identifying differential cis regulatory elements in two cells, they identify TFs that are associated with overexpression or repression of genes of interest. However, this approach of relying solely upon nearest genes of CREs is very cursory and the authors could have used methods such as Activity-by-Contact to establish CREs and their target genes and then assessed their correlation with expression (optional).*

Activity-by-Contact incorporation would be an exciting inclusion in the data analysis, and for the next step in GRN modelling. However, this is out of the scope of this manuscript. In addition, we would like to point out that we did not solely rely on the method of mapping CREs to the nearest genes. Instead, for H3K27ac and ATAC-seq signals, our analysis uses a weighted TSS distance method, within windows of up to 100kb, similar to the method ANANSE and other published gene regulatory network tools. For H3K4me3 and H3K27me3 marks which correlate far better with an expression of the closest genes, we use a window of 2kb at the closest gene.

Reviewer #2:

5. *When ATAC-seq was combined with histone modification analyses, about a third of these regions showed different characteristics, inferring tissue-specific activities. These data may be valuable for identifying tissue-specific cis-regulatory elements (CREs) for the key TFs. This, however, remains to be examined experimentally.*

It is not fully clear to us what ‘experimentally examination’ was referred to by this reviewer, whether to test these tissue-specific CREs individually or globally. We agree that it is important

to test tissue-specific CREs experimentally to examine their function, e.g., which genes they are regulating, and which role they play in tissue-specificity. However, this is out of the scope of this manuscript.

Revision Plan

Reviewer #1 (Evidence, reproducibility and clarity (Required)):

Multi-omics analyses identify transcription factor interplay in corneal epithelial fate determination and disease
The authors of this manuscript describe their work on identifying transcriptional regulators and their interplay in cornea using limbal stem cells and epidermic using keratinocytes. This is a very well written and well described comprehensive manuscript. The authors performed various analyses which were in line with logical workflow of the research question. The authors begin by first identifying differential gene expression signals for the two tissues along with enriched biological processes using GSEA and PROGENy. The strength of this manuscript also includes usage of epigenetic data to determine the cell fate and its drivers. The authors study various epigenetic assays and correlate them expression levels of TFs and genes to identify regulatory patterns that mark differences between LSCs and KCs. By identifying differential cis regulatory elements in two cells, they identify TFs that are associated with overexpression or repression of genes of interest. However, this approach of relying solely upon nearest genes of CREs is very cursory and the authors could have used methods such as Activity-by-Contact to establish CREs and their target genes and then assessed their correlation with expression (optional).
In logical progression, the authors use gene regulatory networks using to compare LSC and KC with Embryonic Stem Cells (ESC) to identify most influential TFs to differentiate them. Along with identifying key TFs, they also identify TF regulatory hierarchy to find TFs that regulate other TFs in context-specific manner. They identify "p63, FOSL2, EHF, TFAP2A, KLF5, RUNX1, CEBPD, and FOXC1 are among the shared epithelial TFs for both LSCs and KCs. PAX6, SMAD3, OTX1, ELF3, and PPARD are LSC specific TFs for the LSC fate, and HOXA9, IRX4, CEBPA, and GATA3 were identified as KC specific TFs." And "p63, KLF4 and TFAP2A can potentially co-regulate PAX6 in LSCs." To compare in vitro findings with in vivo results, they also generate single cell data and identify specific TFs that may play pathobiological role in disease development and progression. While the study is complete and describes well, a strong conclusion, including validation of role of some TFs such as FOSL2 through knock out experiments in model organisms or cell culture will elevate the paper more (optional).
This study merits publication in high quality journal (IF:10-15)

Reviewer #1 (Significance (Required)):

The study is very significant not only in the context of corneal disease biology. The imbalance in interplay of TFs is often envisaged behind disease development but very few efforts of detailed analysis are undertaken. This study is performed very well and the methods are described in clear manner. Appropriate statistical methods are used where required.

Reviewer #2 (Evidence, reproducibility and clarity (Required)):

The study by Smits et al. presents detailed multi-omics (transcripts, ATAC-seq, histone marks)

analyses comparing human limbal stem cells (LSCs) to skin keratinocytes (KCs). The authors compared these two cell types because they have a shared origin in the epidermal progenitors and because LSC diseases occasionally accompany a transition to KC-like phenotypes. The authors analyzed the "omics" data using several bioinformatic analysis tools. Their analyses resulted in a detailed list of the critical transcription factors (TFs) and their gene regulatory networks shared between the two lineages and those unique to either LSCs or KCs.

The findings provide an overview of transcriptional regulators and targets in two essential tissues. This is a valuable tool for future discoveries regarding the processes governing cell differentiation and those involved in the disease mechanism of the cornea. Although the presented predictions are interesting, what is missing is an examination of the functional significance of the findings. Also, as detailed below, there is a need to clarify the source of the cells used for the different analyses.

Comments and suggestions:
1. In Figure 1, the authors compare the transcriptome and epigenome (ATAC-seq and histone modifications) of basal KCs from skin donors and cultured LSCs established from limbal biopsies. The authors should clarify the source of the cells in the published studies - specifically, why more data were needed and if these were comparable to their datasets.
2. Next, they compared the transcriptomes of LSCs and KCs to the transcription profile of LSCs from two aniridia patients and control. They need to specify the stage of the donors' disease and provide details on the control samples. In addition, and as indicated above, when combining published datasets, one should clarify whether the methods of collecting/growing the cells and the disease stage are comparable. This is important with samples from aniridia as it is unclear if the patient LSCs survive the isolation or if other cells take over. The finding that LSC genes are reduced in aniridic LSCs may suggest that the cells resemble KCs, although specific KC genes are not elevated.
3. Figure 2: The authors characterized the regulatory regions in the two cell types based on ATAC-seq and histone marks. Based on ATAC-seq, 80% of the open areas were shared between the two lineages. When ATAC-seq was combined with histone modification analyses, about a third of these regions showed different characteristics, inferring tissue-specific activities. These data may be valuable for identifying tissue-specific cis-regulatory elements (CREs) for the key TFs. This, however, remains to be examined experimentally.
4. It would be valuable to the community if the presented data were also provided online in a web tool so that CRE activity or gene expression could be easily examined.
5. Using motif predictions, the authors point to the TF families that likely control the differential CREs (Figure 3). Next, the authors constructed the gene regulatory network based on the (ANANSE) pipeline, which integrates CRE and TF motif predictions with the expression of TFs and their target genes. To gain further insight into the shared gene regulatory networks, they compared each to similar data from embryonic stem cells. Their analysis further suggests shared TFs regulating each other and some of the tissue-specific transcription factors. Differential gene expression of the TFs was partially validated by analyzing available single-cell data (Figure 4).

6. In the final section of the study, the authors aimed to identify TFs in LSCs that are relevant to corneal disease. They examined whether the LSC TFs are bound to genes associated with LSC deficiency and inherited corneal diseases. To accomplish this task, the authors incorporated single-cell data on corneal gene expression and available datasets on genetic analyses of families. Through this analysis, they identified a mutation in FOSL2 that may be causing corneal opacity in the carriers. Also, the findings indicate an interaction between FOXL2 and other TFS is important for maintaining the corneal epithelium. These interesting predictions indicate an important role for FoxL2 in corneal function. It would be important to verify these predictions by experimental studies, for example, by presenting the association of FOXL2 with the predicted co-factors and presenting data on the effect of the identified mutation on FOXL2 transcriptional activity.

Reviewer #2 (Significance (Required)):

The analysis provides an overview of transcriptional regulators and targets in two essential tissues. This is a valuable tool for future discoveries regarding the processes governing cell differentiation and those involved in the disease mechanism of the cornea. The results predict a role for FoxL2 in corneal function.
