# Supplementary material for: Identification of the regulatory circuit governing corneal epithelial fate determination and disease
Source: PLoS Biol. 2023 Oct 19;21(10):e3002336. doi: 10.1371/journal.pbio.3002336 (PMC10586658; doi:10.1371/journal.pbio.3002336)
Supplement: Attachment — Submitted filename: PLOS_Bio_response_reviewers.pdf [file pbio.3002336.s025.pdf]

Point to point response to reviewers' comments

Reviewer remarks:

Original Reviewer #1, Abhijeet Sonawane (note, this reviewer has signed their review):
The authors have revised the manuscript substantially and have address the concerns and queries raised by this reviewer. This reviewer is very satisfied with the scientific quality of the manuscript and recommend its acceptance. I would think including PAX6 and FOSL2 relationship in title itself may increase the appeal of the paper.
We thank the reviewer for the kind comments. We have updated the title per the Editor's suggestion.

Original Reviewer #2:
In the revised manuscript, the authors included functional studies of the FOSL2, a gene predicted by their multi-omic analysis to play an essential role in corneal function. The functional validation included mapping the binding sites of FOSL2 by cut-and-run and knockdown approach to identify the targets. The authors also provide additional experimental evidence for regulating FOSL2 by PAX6. The authors addressed the reviewer's concerns on the data analysis and added new data that experimentally substantiates the role of FOSL2 in corneal function.

The study provides an overview of transcriptional regulators and targets in two essential tissues; human limbal stem cells and skin keratinocytes. The multi-omic comparison is valuable for delving into the processes governing cell differentiation and those involved in the disease mechanism of the cornea. Through their analyses and experimental validation, the authors expose the importance of FOXL2 in corneal function.
We thank the reviewer for the positive comments.

New Reviewer #3:
This study generated multi-omics analysis of the corneal epithelium. The authors also compared the CREs between two stratified epithelia, corneal epithelial cells and keratocytes. They found a novel candidate, FOSL2, acts as a downstream transcription factor of PAX6. And this gene may associate with various eye diseases. These datasets offer an important resource for understanding the regulatory mechanism of corneal epithelial fate determination.

The major concern is that the potential function of FOSL2 in corneal epithelial cells found by the authors is related to angiogenesis and tight junction, however, the corneal epithelial diseases studied here is corneal dystrophies and keratoconus, the link between the phenotype and mechanism is pretty week. The focus for current manuscript is corneal epithelial cell fate, the analysis of FOSL2 in glaucoma, FECD, cataract, etc. seems unnecessary and lack of further evidence.
We thank reviewer 3 for the supportive comments. The aim of the analysis is to reinforce the importance of the key transcription factors as regulators of overall cornea health. For this, we used a holistic approach, investigating several key transcription factors including PAX6, p63 and FOSL2 by **predicting** their disease-associated target genes in all eye diseases, using our own curated disease gene list or the EyeDiseases database (ref 57 in the manuscript). In this EyeDiseases database, there are more genes associated with common diseases such as glaucoma, and less genes for specific epithelial eye diseases. Although not yet proven with functional data, our analysis clearly indicates the important role of FOSL2 in eye diseases. Regarding 'angiogenesis ad tight junction' mentioned in the manuscript, they are the functional terms detected among differential genes upon FOSL2 knock-down (see below). This clearly shows that loss of FOSL2 in limbal epithelial cells gives rise to non-epithelial characteristics, demonstrating the importance of FOSL2 in epithelial cells and in related

diseases. To avoid confusion, we have shortened and clarified the rationale and the findings in Results (lines 447-448) and in Discussion (line 613-622).

The authors should identify the role of FOSL2 in real corneal epithelial diseases and verify the pathological changes.

The rationale to perform functional analysis of FOSL2 shown in Fig.6 is because a likely damaging variant in FOSL2 was identified in a patient with corneal opacity. By performing CUT&RUN and siRNA knockdown experiments specifically in limbal epithelial stem cells, we demonstrate FOSL2 is indeed paramount for epithelial fate, by i) repressing vascularisation genes - avascularity is an important feature of corneal epithelium health; and 2) deregulating tight junction genes expression, which are essential for barrier function, a hallmark of epithelial cells. All data combined, we argue that FOSL2 defects can indeed cause corneal disease. To demonstrate the effect of the genetic variant in the disease is out of the scope for this manuscript, as we discussed with other two reviwers in the previous rebuttal.

We mention this in Discussion (lines 636-651).

Additional comment ：FOSL2 is expressed in both LSCs and KCs. Does FOSL2 function differently in KCs?

We showed the presence of FOSL2 in both cell types by fluorescence stainings to confirm our prediction that FOSL2 is a shared TF. Although interesting, we did not investigate its role in KCs, as the focus of this study sat on corneal fate and disease (Discussion, lines 650-651).
