# Supplementary material for: Identification of the regulatory circuit governing corneal epithelial fate determination and disease
Source: PLoS Biol. 2023 Oct 19;21(10):e3002336. doi: 10.1371/journal.pbio.3002336 (PMC10586658; doi:10.1371/journal.pbio.3002336)
Supplement: Attachment — Submitted filename: Point2Point_response_V2.docx [file pbio.3002336.s024.docx]

**Point-to-point response**

**Reviewer #1 Evidence, reproducibility and clarity:**
*Multi-omics analyses identify transcription factor interplay in corneal epithelial fate determination and disease
The authors of this manuscript describe their work on identifying transcriptional regulators and their interplay in cornea using limbal stem cells and epidermic using keratinocytes. This is a very well written and well described comprehensive manuscript. The authors performed various analyses which were in line with logical workflow of the research question. The authors begin by first identifying differential gene expression signals for the two tissues along with enriched biological processes using GSEA and PROGENy. The strength of this manuscript also includes usage of epigenetic data to determine the cell fate and its drivers. The authors study various epigenetic assays and correlate them expression levels of TFs and genes to identify regulatory patterns that mark differences between LSCs and KCs. By identifying differential cis regulatory elements in two cells, they identify TFs that are associated with overexpression or repression of genes of interest. However, this approach of relying solely upon nearest genes of CREs is very cursory and the authors could have used methods such as Activity-by-Contact to establish CREs and their target genes and then assessed their correlation with expression (optional).*

Using the Activity-by-Contact approach to establish CREs and their target genes would be an exciting inclusion in the data analysis and for the next step in GRN modeling. However, the computational tool applied in the manuscript such as ANANSE does include this option. To perform this analysis, we would need to develop/include new functions in ANANSE; therefore, this is out of the scope of this manuscript. In addition, we did not solely rely on the method of mapping CREs to the nearest genes. Instead, for H3K27ac and ATAC-seq signals, our analysis uses a weighted TSS distance method, within windows of up to 100kb, similar to ANANSE and other published gene regulatory network tools. For H3K4me3 and H3K27me3 marks which correlate far better with the expression of the closest genes, we use a window of 2kb at the closest gene. For the disease gene enrichment, we include both a ‘mapping to the closest gene’ approach and a peak distance weight Man Whitney U test based on the distance weight method.

*In logical progression, the authors use gene regulatory networks using to compare LSC and KC with Embryonic Stem Cells (ESC) to identify most influential TFs to differentiate them. Along with identifying key TFs, they also identify TF regulatory hierarchy to find TFs that regulate other TFs in context-specific manner. They identify "p63, FOSL2, EHF, TFAP2A, KLF5, RUNX1, CEBPD, and FOXC1 are among the shared epithelial TFs for both LSCs and KCs. PAX6, SMAD3, OTX1, ELF3, and PPARD are LSC specific TFs for the LSC fate, and HOXA9, IRX4, CEBPA, and GATA3 were identified as KC specific TFs." And "p63, KLF4 and TFAP2A can potentially co-regulate PAX6 in LSCs." To compare in vitro findings with in vivo results, they also generate single cell data and identify specific TFs that may play pathobiological role in disease development and progression. While the study is complete and describes well, a strong conclusion, including validation of role of some TFs such as FOSL2 through knock out experiments in model organisms or cell culture will elevate the paper more (optional).*

To address this point, we have followed the suggestion of the reviewer and performed siRNA knockdown experiments of FOSL2 and PAX6 in primary LSCs. We examined the transcriptional consequences of knocking down these TFs by RNA-seq and RT-qPCR analyses. Importantly we identified a major repressive role of FOSL2 of angio-genesis-related genes, as well as its role in regulating tight junction genes. Furthermore, we identified that *FOSL2* is a direct PAX6 target, strengthening its important role in LSCs.

This study merits publication in high quality journal (IF:10-15)

**Reviewer #1 Significance:**
The study is very significant not only in the context of corneal disease biology. The imbalance in interplay of TFs is often envisaged behind disease development but very few efforts of detailed analysis are undertaken. This study is performed very well and the methods are described in clear manner. Appropriate statistical methods are used where required.

**Reviewer #2 Evidence, reproducibility and clarity ):**
*The study by Smits et al. presents detailed multi-omics (transcripts, ATAC-seq, histone marks) analyses comparing human limbal stem cells (LSCs) to skin keratinocytes (KCs). The authors compared these two cell types because they have a shared origin in the epidermal progenitors and because LSC diseases occasionally accompany a transition to KC-like phenotypes. The authors analyzed the "omics" data using several bioinformatic analysis tools. Their analyses resulted in a detailed list of the critical transcription factors (TFs) and their gene regulatory networks shared between the two lineages and those unique to either LSCs or KCs.

The findings provide an overview of transcriptional regulators and targets in two essential tissues. This is a valuable tool for future discoveries regarding the processes governing cell differentiation and those involved in the disease mechanism of the cornea. Although the presented predictions are interesting, what is missing is an examination of the functional significance of the findings.*

We validated the importance of the key TFs with three different approaches. As FOSL2 is the novel disease candidate factor identified in this study, we have given more emphasis on its functional validation. First of all, we performed immunostaining of FOSL2, FOSL1, PAX6 & TP63 in the human cornea and in vitro cultured LSCs and KCs, showing clear expression of all these factors in expression in the human corneal epithelium and in *in vitro* cultured LSCs. Secondly, we performed FOSL2 CUT&RUN to identify its binding sites in the genome and to predict its target genes. This also allowed us to identify an enrichment of FOSL2 binding in genes associated with eye and cornea diseases. Finally, we performed siRNA experiments for FOSL2 and PAX6, followed by RNA-seq and RT-qPCR analysis. These experiments showed that PAX6 regulates *FOSL2* expression, and FOSL2 represses angiogenesis and tight junction genes. All these additional functional validation experiments demonstrate the importance and significance of the *FOSL2* in cornea biology and diseases.

*Also, as detailed below, there is a need to clarify the source of the cells used for the different analyses.*

We have made detailed comments to address this point below.

*Comments and suggestions:
1. In Figure 1, the authors compare the transcriptome and epigenome (ATAC-seq and histone modifications) of basal KCs from skin donors and cultured LSCs established from limbal biopsies. The authors should clarify the source of the cells in the published studies - specifically, why more data were needed and if these were comparable to their datasets.*

We have included the cell sources and cultured conditions from the published studies and added additional columns in the supplementary tables 1, 2, and 3. Briefly, LSCs reported in published studies were extracted from post-mortem cornea and cultured in DMEM/F12, or KSFM.

Regarding the questions on the necessity of incorporating publicly available data with our own data, our reasoning was two-fold. First, we see this integrated approach as a strength, as the most important differences between cell types that determine cell fates should be consistent in cells generated from different donors and labs. Second, we chose to generate more data in our own lab in order to make sure to have comparisons without the influence of technical differences between publicly available datasets. We include text to clarify these data in the Discussion (lines 535-539). Furthermore, to show that the datasets we used are consistent and can be integrated, we have performed a PCA correlation analysis for the RNA-seq analysis (supplementary figures 1A&1B lines 945-951) and added a spearman correlation analysis for the ATAC-seq datasets (supplementary figure 5F & lines 933-934). Both approaches showed that clear biological signal similarities are more pronounced than the technical variations from different labs and techniques.

*2. Next, they compared the transcriptomes of LSCs and KCs to the transcription profile of LSCs from two aniridia patients and control. They need to specify the stage of the donors' disease and provide details on the control samples. In addition, and as indicated above, when combining published datasets, one should clarify whether the methods of collecting/growing the cells and the disease stage are comparable. This is important with samples from aniridia as it is unclear if the patient LSCs survive the isolation or if other cells take over. The finding that LSC genes are reduced in aniridic LSCs may suggest that the cells resemble KCs, although specific KC genes are not elevated.*

Healthy LSC cells used in the direct comparison with aniridia LSC cells were grown using the same expansion and culture conditions. Furthermore, the methods of culture, extraction, and expansion between the earlier published aniridia cell RNAseq comparison and our data are exactly the same (lines 746:752). Both aniridia samples were from patients of stage 4 on the Lagali Stages (Lagali, N. et al. (2020) ‘Early phenotypic features of aniridia-associated keratopathy and association with PAX6 coding mutations’, The Ocular Surface, 18(1), pp. 130–140. We have included this information in Material and Methods (lines 743-744). For healthy control cells, no information regarding the stage and gender is available, as they are from anonymous individuals. We added more information on the aniridia and control samples regarding the culture conditions and passage numbers (lines 751:752). We have included the cell sources from the aniridia samples and added additional columns in supplementary table 1. We like to again emphasize that including publicly available data, together with our in-house data, even though they are derived from different donor or patient groups in different research labs, strengthens our conclusion that the similarities and differences between controls and aniridia patients do not result from technical variations from different labs and techniques.

*3. Figure 2: The authors characterized the regulatory regions in the two cell types based on ATAC-seq and histone marks. Based on ATAC-seq, 80% of the open areas were shared between the two lineages. When ATAC-seq was combined with histone modification analyses, about a third of these regions showed different characteristics, inferring tissue-specific activities. These data may be valuable for identifying tissue-specific cis-regulatory elements (CREs) for the key TFs. This, however, remains to be examined experimentally.*

It is not fully clear to us what ‘experimentally examination’ was referred to by this reviewer, whether to test these tissue-specific CREs individually or globally. We agree that it is important to test tissue-specific CREs experimentally to examine their function, e.g., which genes they are regulating, and which role they play in tissue-specificity. However, this is out of the scope of this manuscript.

*4. It would be valuable to the community if the presented data were also provided online in a web tool so that CRE activity or gene expression could be easily examined.*

We fully agree with this reviewer that our data should be shared with the community. In addition to having deposited our data in the GEO database and the our analysis pipelines in github which are freely accessible, we have expanded the UCSC track hub to highlight the identified variable CRE elements. This will enable a searchable tool for differential CREs close to genes of interest between KCs and LSCs. We have added a sentence to explicitly mention the presence of this track hub in the result section (lines 254-255).

*5. Using motif predictions, the authors point to the TF families that likely control the differential CREs (Figure 3). Next, the authors constructed the gene regulatory network based on the (ANANSE) pipeline, which integrates CRE and TF motif predictions with the expression of TFs and their target genes. To gain further insight into the shared gene regulatory networks, they compared each to similar data from embryonic stem cells. Their analysis further suggests shared TFs regulating each other and some of the tissue-specific transcription factors. Differential gene expression of the TFs was partially validated by analyzing available single-cell data (Figure 4).*  *6. In the final section of the study, the authors aimed to identify TFs in LSCs that are relevant to corneal disease. They examined whether the LSC TFs are bound to genes associated with LSC deficiency and inherited corneal diseases. To accomplish this task, the authors incorporated single-cell data on corneal gene expression and available datasets on genetic analyses of families. Through this analysis, they identified a mutation in FOSL2 that may be causing corneal opacity in the carriers. Also, the findings indicate an interaction between FOXL2 and other TFS is important for maintaining the corneal epithelium. These interesting predictions indicate an important role for FoxL2 in corneal function. It would be important to verify these predictions by experimental studies, for example, by presenting the association of FOXL2 with the predicted co-factors and presenting data on the effect of the identified mutation on FOXL2 transcriptional activity.*

We assume that Reviewer #2 refers to FOSL2 instead of FOXL2. We agree with this reviewer’s suggestion to functionally address the importance of *FOSL2* in the cornea, as the function of *FOSL2* in the cornea is completely unknown. Same as addressed above, in order to answer this, we first performed FOSL2 staining in the human cornea, as well as in cultured LSCs. Second, we performed FOSL2 CUT&RUN to identify FOSL2 binding and identify its target genes. Finally, we performed siRNA experiments for FOSL2 and PAX6, to identify the consequence of their deregulation and their regulatory hierarchy. Regarding the clinical effect of the specific *FOSL2* variant reported in our study, functional validation would strengthen our work even more. However, the study’s main message is integrative omics analyses to uncover new transcription factors involved in corneal/limbal fate and to highlight new candidate genes in corneal disease. Therefore, we feel that the disease mechanism behind the specific *FOSL2* variant, albeit interesting, is beyond the scope of this study. Nonetheless, we reinforced the pathogenicity of the variant with various in silico predictions (supplementary table 9). Interestingly, a recent study reported that *FOSL2* truncation mutations are involved in a new syndrome with congenital ectodermal defects and cataracts. This aligns with our findings that FOSL2 is an important shared TF in both LSCs and KCs, strengthening the predicted role of FOSL2 in the epithelium of the eye and associated diseases. We have included additional discussion on this and other FOSL2-related studies in the Discussion.

***Reviewer #2 Significance:***  *The analysis provides an overview of transcriptional regulators and targets in two essential tissues. This is a valuable tool for future discoveries regarding the processes governing cell differentiation and those involved in the disease mechanism of the cornea. The results predict a role for FoxL2 in corneal function.*
